# Screening and contact precautions – A survey on infection control measures for multidrug-resistant bacteria in German university hospitals

**DOI:** 10.1186/s13756-017-0191-2

**Published:** 2017-04-13

**Authors:** Lena M. Biehl, Hartmut Bertz, Johannes Bogner, Ute-Helke Dobermann, Johanna Kessel, Carolin Krämer, Sebastian Lemmen, Marie von Lilienfeld-Toal, Silke Peter, Mathias W. Pletz, Holger Rohde, Stefan Schmiedel, Sören Schubert, Andrew J. Ullmann, Gerd Fätkenheuer, Maria J. G. T. Vehreschild

**Affiliations:** 1 Department I of Internal Medicine, University Hospital of Cologne, Kerpener Strasse 62, 50937 Cologne, Germany; 2 Department of Haematology/Oncology and Stem Cell Transplantation, University Medical Centre, Freiburg, Germany; 3 Department of Infectious Disease, Med IV, University Hospital of Munich, Munich, Germany; 4 Center for Infectious Diseases and Infection Control, Jena University Hospital, Jena, Germany; 5 Infectious Diseases, Medical Clinic II, Johann Wolfgang Goethe-University Frankfurt, Frankfurt, Germany; 6 Department of Haematology, Oncology, Haemostaseology and Stem Cell Transplantation, Medical Faculty, RWTH Aachen University Hospital, Aachen, Germany; 7 Division of Infection Control and Infectious Diseases, Medical Faculty, RWTH Aachen University Hospital, Aachen, Germany; 8 Department of Internal Medicine II, Haematology and Medical Oncology, Jena University Hospital, Jena, Germany; 9 Institute of Medical Microbiology and Hygiene, University of Tübingen, Tübingen, Germany; 10 Institute for Medical Microbiology, Virology and Hygiene, University Medical Centre Hamburg-Eppendorf, Hamburg, Germany; 11 1st Department of Medicine, University Medical Centre Hamburg–Eppendorf, Hamburg, Germany; 12 Max-von-Pettenkofer Institute, Ludwig-Maximilians-University, Munich, Germany; 13 Department of Internal Medicine II, Division of Infectious Diseases, University Hospital Würzburg, Würzburg, Germany; 14 German Centre for Infection Research (DZIF), site Bonn-Cologne, Cologne, Germany; 15 German Centre for Infection Research (DZIF), Tübingen, Germany

**Keywords:** Multidrug-resistant bacteria, Infection control, Contact precautions, Colonisation

## Abstract

To assess the scope of infection control measures for multidrug-resistant bacteria in high-risk settings, a survey among university hospitals was conducted. Fourteen professionals from 8 sites participated. Reported policies varied largely with respect to the types of wards conducting screening, sample types used for screening and implementation of contact precautions. This variability among sites highlights the need for an evidence-based consensus of current infection control policies.

**Electronic supplementary material:**

The online version of this article (doi:10.1186/s13756-017-0191-2) contains supplementary material, which is available to authorized users.

## Background

In the light of increasing rates of antibiotic resistance worldwide, there is an ongoing discussion on the adequacy of specific infection control strategies in preventing transmission of multidrug-resistant bacteria. Namely, extended-spectrum ß-lactamase producing Enterobacteriaceae (ESBL-E), vancomycin-resistant *Enterococci* (VRE) and methicillin-resistant *Staphylococcus aureus* (MRSA) are subject of various studies in the field evaluating screening policies, contact precautions and decolonisation practices. Yet, results are often contradictory and the evidence regarding the effectiveness of different infection control measures remains inconclusive [1–3]. In Germany, infection control guidelines of the Commission for Hospital Hygiene and Infection Prevention at the Robert Koch-Institute (KRINKO) exist for MRSA [4] and ESBL-E [5], but not VRE. However, most of the respective recommendations are based on expert opinions only and in the case of ESBL-E the German guidelines differ to those from the European Society of Clinical Microbiology and Infectious Diseases (ESCMID) in some aspects [6].

In clinical practice, these discrepancies together with organisational and economic constraints lead to a high variability in the actual implementation of infection control measures. The present survey aimed at approaches currently established in high-risk wards in German tertiary hospitals.

## Methods

A survey was conducted between June and October 2013 assessing the current practice in screening and contact precautions for extended-spectrum ß-lactamase producing Enterobacteriaceae (ESBL-E), vancomycin-resistant *Enterococci* (VRE) and methicillin-resistant *S. aureus* (MRSA) on adult intensive care units (ICUs) and haematological/oncological wards (HO), which are usually categorized as high risk units with regard to acquisition of multidrug-resistant organisms. Of note, a new nomenclature of multi-drug resistant gramnegative bacteria in relation with infection control recommendations was introduced by the German commission of hospital hygiene and infection prevention (KRINKO) during the survey design [5]. This nomenclature distinguishes between gram negatives with resistance to 3 (3 MRGN) or 4 antibiotic classes (4 MRGN) without taking the responsible resistance mechanism into account. This terminology was not used in our survey and participants were asked to report management of 3MRGN as ESBL-E, even though a quinolone susceptible ESBL-E is not regarded a 3MRGN. Furthermore, due to relatively low absolute numbers of carbapenem-resistant bacteria in Germany during the time of study conduct [7], we did not address them in our survey. Additionally, in the majority of cases, these organisms are detected by ESBL-E screening and there is no controversy on the necessity of strict contact precautions.

The survey was designed as an online questionnaire using the software Questback EFS Survey with personalized links sent out to the participants. An English language print-version is available as Additional file 1. The extent of screening samples analysed per site was assessed retrospectively via mail.

Overall, 23 physicians from 10 different university hospitals were contacted to participate addressing professionals from at least two different specialities and departments (i.e., Infectious Diseases, Microbiology and Hygiene, Haematology and Oncology). The selection of university hospitals was based on the presence of all mentioned departments. In case of discrepant statements from the professionals of the same hospital, the respective participants were asked to agree on a common answer in order to allow comparison between sites.

## Results

### Participants

Fourteen professionals from 8 hospitals completed the questionnaire. In all 5 cases with more than one participant from the same hospital, discrepant responses were provided to at least one question. Discrepancies were discussed among the respective participants, and an agreement on one common answer was achieved in all cases.

### Screening policies

The participants were asked which wards perform an admission screening for ESBL-E, MRSA and VRE and regular follow-up screening during inpatient stay irrespective of previously detected colonisation. All three pathogens are targeted during admission screening in 5 hospitals, two hospitals target only MRSA and one hospital only VRE. The only pathogen screened for on hospital level is MRSA (5 of 8 hospitals), while ESBL-E and VRE are mainly screened for in HO (5 hospitals) and less frequently ICU (2 hospitals). Follow-up screenings on different wards are established in 5 of 8 hospitals.

In a second step, the participants were asked for specific details of screening implementation. Admission screening is performed within 48 h and follow-up screening on a weekly basis, irrespective of the pathogen in all hospitals with these procedures established. ESBL-E and VRE screening consistently involves all patients admitted to the wards irrespective of risk-factors. Two hospitals screen all patients for MRSA admitted to certain high-risk wards only, 5 hospitals screen patients with specific risk-factors [4] hospital wide, and one hospital does not screen for MRSA.

There is a high variability in the sample techniques used for screening. As expected, rectal swabs are included in all sample sets, except one hospital using a perianal swab for VRE, instead. Sampled sites for ESBL-E and VRE are identical between admission and follow-up screening. The detailed sets for ESBL-E and VRE screening are shown in Table 1.

**Table 1:** Sets of samples used in screening for ESBL-E and VRE

Combination of samples	ESBL-E screening - no. of hospitals (*N* = 5)	VRE screening - no. of hospitals (*N* = 6)
Only rectal swab	2	2
Only rectal swab or stool sample	2	1
Only perianal swab	0	1
Perianal swab and stool sample	0	1
Rectal swab, throat swab and urine	1	0
Rectal swab and urine	0	1

*ESBL-E* extended-spectrum ß-lactamase producing Enterobacteriaceae, *VRE* vancomycin-resistant *Enterococci*

All seven hospitals screening for MRSA use nose swabs. For admission screening, six different combinations of samples were reported: only nose swab (one hospital); swabs from nose, groin and axilla (1); swabs from nose, groin and wounds (1); swabs from nose, wounds and previously colonised sites (2); swabs from nose, groin, axilla and throat (1); swabs from nose, groin, axilla and wound (1). Only nasal swabs are used in the two hospitals performing regular follow-up screening. Microbiological detection of MRSA involves PCR-based testing in addition to culture in four sites, the remaining three sites use only culture.

Overall, the above described policies lead to a high number of screenings performed in the respective microbiological laboratories. The mean number of screenings performed in 2013 for ESBL-E was 7128 samples (range 2410–14,905), for VRE 5749 samples (range 1105–9472) and for MRSA 4605 samples (range 684–9136).

### Contact precautions

Overall, four different policies of contact precautions were reported. Half of the participating sites (four hospitals) apply contact precautions for all three pathogens throughout the hospital, two apply them for all pathogens but only in ICUs and HO wards, one site applies contact precautions for VRE and MRSA on a hospital-wide level and one reported applying them for VRE and MRSA in the whole hospital but for ESBL-E only on ICUs and HO wards.

The rules for accommodating colonised patients and for entrance to their rooms are illustrated in Table 2. For ESBL-E and VRE the most frequently reported contact precautions combine accommodation of colonised patients in single rooms or cohort isolation plus use of gloves and gowns when entering the room. Regarding MRSA, the use of face masks in addition to these precautions is applied. Of note, one hospital does not apply any contact precautions for patients with ESBL-E and another hospital does only apply wearing of gloves and gowns without single room accommodation in cases with ESBL-E or VRE.

**Table 2:** Elements of contact precautions according to colonising pathogen

Contact precautions	ESBL-E (*N* = 7)	VRE (*N* = 8)	MRSA (*N* = 8)
Single room or cohorting, gloves, gowns, masks and hair cover	1	0	2
Single room or cohorting, gloves, gowns and masks	1	2	3
Single room or cohorting, gloves and gowns	4^a^	3^a^	3
Single room or cohorting, gowns	0	1	0
Only single room or cohorting	0	1	0
Only gloves and gowns	1	1	0

*ESBL-E* extended-spectrum ß-lactamase producing Enterobacteriaceae, *VRE* vancomycin-resistant *Enterococci*, *MRSA* methicillin-resistant *S. aureus*

^a^One site additionally stated, that masks are applied in case of colonisation with ESBL-E or VRE in the upper respiratory tract

Furthermore, we assessed the conditions under which colonised patients were allowed to leave their room and the respective contact precautions taken. Participants from most sites (6 hospitals) stated that colonised patients regardless of pathogen are allowed to leave their room at any time provided that certain precautions are taken, while in one hospital leaving the room was only allowed for urgent diagnostics; one hospital was unsure about the conditions. The reported measures varied from gowning only (1 hospital for ESBL-E, 2 for VRE) to wearing of gloves and gowns (4 hospitals for ESBL-E and VRE each, 2 for MRSA) or additional wearing of masks (MRSA only, 4 hospitals).

### Further infection control measures

Six hospitals perform decolonisation of MRSA in all colonised patients, while two restrict this to certain patient groups, i.e., to those with “realistic eradication chances” (one hospital) and those “without tracheotomy and without extensive colonisation” (one hospital).

Finally, the participants were asked about adaption of empirical antibiotic treatment in the case of supposed infection in colonised patients. The majority (six hospitals) adjusts their antibiotic regimen to the colonisation status in relation to all three pathogens, one hospital adjusts only in case of ESBL-E and one stated to have no clear strategy.

## Discussion

This survey addressed the practice of screening for multidrug-resistant bacteria and infection control measures of haematological/oncological wards and intensive care units of German university hospitals. The reported policies of participating sites differed widely in many aspects, and even among participants from individual sites, there were inconsistencies between answers. The latter observation is even more surprising since all participants had a strong background in infectious diseases.

The number of screenings performed showed that screening for multi-resistant bacteria is performed very frequently with several thousands of samples being analysed annually per site. All sites performed some kind of admission screening within 48 h on the high-risk wards and five of eight sites have implemented follow-up screening. However, the detailed implementation of screening varied considerably. Similarly, four different policies regarding wards applying contact precautions and at least three different combinations of elements for contact precautions for each pathogen reported were reported in the questionnaire. Particularly regarding ESBL-E the reported contact precautions ranged from none to single room accommodation plus wearing of gloves, gowns, masks and hair cover.

While this questionnaire cannot give a representative picture of the infection control measures in German university hospitals, it clearly shows that there is hardly any consensus among different sites despite existing guidelines for the management of MRSA and ESBL-E and despite the large dimension of screenings actually performed. It is highly likely that this variability would be equally apparent in a larger study as previously reported [8, 9]. One can think of a number of reasons for this: First of all, the local epidemiology may trigger certain infection control measures to be implemented or omitted in deviation from the guidelines. Secondly, the complexity of many recommendations promotes different interpretations of existing standards in clinical practice – probably also explaining the inconsistencies within one institution. Thirdly, the evidence for infection control measures is contradictory in the case of MRSA [10] and scarce in the case of ESBL-E and VRE [2, 3] undermining guideline adherence.

The presented differences in infection control measures highlight the need for guidelines that have the potential for broad acceptance in the medical community. Different management of patients colonised or infected with multidrug- resistant organisms in hospitals or even on different wards of the same hospital may lead to severe perturbation of patients. Given the increasing prevalence of patients colonised or infected by multidrug-resistant bacteria worldwide, practical issues are becoming more relevant than ever: A higher proportion of patients in contact isolation has been reported to be associated with a lower compliance with precautions [11]. The increase in carbapenem-resistant organisms also in German high-risk settings [7], may urge one to prioritize infection control measures. Moreover, contact precautions are known to be associated with negative effects to patients’ wellbeing and patient management [12], and therefore need good evidence and strong indications for implementation. Well-designed clinical studies evaluating infection control measures and taking the above mentioned practical issues into account should facilitate future guideline development. While we did not assess management of carbapenem-resistant organisms in this survey, a joint and evidence-based approach is of even higher importance for those organisms with very few therapeutic options left.

In conclusion, our study demonstrates a high variability of the use of infection control measures among different hospitals and among different physicians within the same hospital. Simple, evidence based and consented guidelines are needed for better guidance of health care providers in the use of infection control practices.

## Additional file

Additional file 1: English language print-version of survey. (PDF 123 kb)

## Abbreviations

ESBL-E: Extended-spectrum ß-lactamase producing Enterobacteriaceae
HO: Haematological and oncological wards
ICU: Intensive care unit
MRSA: Methicillin-resistant *Staphylococcus aureus*
VRE: Vancomycin-resistant *Enterococci*
