# Supplementary material for: Screening and contact precautions – A survey on infection control measures for multidrug-resistant bacteria in German university hospitals
Source: Antimicrob Resist Infect Control. 2017 Apr 13;6:37. doi: 10.1186/s13756-017-0191-2 (PMC5390437; doi:10.1186/s13756-017-0191-2)
Supplement: Additional file 1: — English language print-version of survey. (PDF 123 kb) [file 13756_2017_191_MOESM1_ESM.pdf]

Screening and contact precautions – A survey on infection control measures for multidrug-resistant bacteria in German university hospitals

Supplementary Material: English-language print version of survey

---

Participant-Details

Hospital city: ____

Name of hospital: ____

Name of department: ____

Which specialty is this department

- ☐ Microbiology/hygieen
- ☐ Haematology/Oncology
- ☐ Infectious Disease
- ☐ Intensive Care
- ☐ Other specialty, please indicate: ________________________________

1. Screening on resistant pathogens in your hospital

The following question refer to the departments:

- Haematology/Oncology
- Intensive Care Units

Later on, you will have the possibility to state whether the whole hospital has the identical approach.

a. Which pathogens are screened for after admission to these wards? – *Multiple answers possible*

☐ ESBL producing Enterobacteriaceae (ESBL)

☐ Vancomycin-resistant *Enterococci* (VRE)

☐ Methicillin-resistant *Staphylococcus aureus* (MRSA)

☐ No admission screening on these wards

☐ Unknown or uncertain

b. Which pathogens are screened for on a follow-up basis during inpatient stay on these wards?
– This refers to regular screenings not part of the admission screening. Multiple answers possible

☐ ESBL

☐ VRE

☐ MRSA

☐ No regular follow-up screening on these wards

☐ Unknown or uncertain

2. Admission screening for ESBL

a. Which wards perform admission screening for ESBL? - *Multiple answers possible*

- ☐ Haematology/Oncology
- ☐ Intensive Care Units
- ☐ Whole hospital (only if screening approach is identical in the whole hospital)
- ☐ Unknown or uncertain

b. Which patients are screened on admission to these wards?

- ☐ All patients
- ☐ Patients with known colonisation
- ☐ Other patients with a certain risk – please indicate: ______________________________________
- ☐ Unknown or uncertain

c. How is ESBL sceening performed? - *Multiple answers possible*

- ☐ Rectal swab
- ☐ Stool sample
- ☐ Rectal swab or stool sample
- ☐ Perianal swab
- ☐ Throat swab
- ☐ Urine sample
- ☐ Other, please indicate: ___________________________________
- ☐ Unknown or uncertain

d. When is ESBL admission screening performed?

- ☐ Within 48 hours of admission
- ☐ Within 72 hours of admission
- ☐ Other, please indicate: ____
- ☐ Unknown or uncertain

e. Are patients pre-emptively put under contact precautions until results of the screening are available?

- ☐ Yes
- ☐ No
- ☐ Only in case of previous colonisation.
- ☐ Unknown or uncertain

f. Comments on admission screening

In case the above details do not reflect your hospital’s approach correctly, please indicate further details:

3. ESBL-Screening during inpatient stay

a. Which wards perform regular follow-up screening for ESBL? - *Multiple answers possible*

- ☐ Haematology/Oncology
- ☐ Intensive Care Units
- ☐ Whole hospital (only if screening approach is identical in the whole hospital)
- ☐ Unknown or uncertain

b. Which patients are screened during inpatient stay in these wards?

- ☐ All patients
- ☐ Patients with known colonisation
- ☐ Other patients with a certain risk – please indicate:
  ____
- ☐ Unknown or uncertain

c. How is ESBL sceening performed? - *Multiple answers possible*

- ☐ Rectal swab
- ☐ Stool sample
- ☐ Rectal swab or stool sample
- ☐ Perianal swab
- ☐ Throat swab
- ☐ Urine sample
- ☐ Other, please indicate: ___________________________________
- ☐ Unknown or uncertain

d. How often is ESBL follow-up screening performed?

- ☐ Once per week
- ☐ Shortly before discharge
- ☐ Other frequency, please indicate: ____
- ☐ Unknown or uncertain

e. Comments on follow-up screening

In case the above details do not reflect your hospital's approach correctly, please indicate further details:

4. Admission screening for VRE

a. Which wards perform admission screening for VRE? - *Multiple answers possible*

- ☐ Haematology/Oncology
- ☐ Intensive Care Units
- ☐ Whole hospital (only if screening approach is identical in the whole hospital)
- ☐ Unknown or uncertain

b. Which patients are screened on admission to these wards?

- ☐ All patients
- ☐ Patients with known colonisation
- ☐ Other patients with a certain risk – please indicate:

  ____

- ☐ Unknown or uncertain

c. How is VRE sceening performed? - *Multiple answers possible*

- ☐ Rectal swab
- ☐ Stool sample
- ☐ Rectal swab or stool sample
- ☐ Perianal swab
- ☐ Throat swab
- ☐ Urine sample
- ☐ Other, please indicate: ___________________________________
- ☐ Unknown or uncertain

d. When is VRE admission screening performed?

- ☐ Within 48 hours of admission
- ☐ Within 72 hours of admission
- ☐ Other, please indicate: ____
- ☐ Unknown or uncertain

e. Are patients pre-emptively put under contact precautions until results of the screening are available?

- ☐ Yes
- ☐ No
- ☐ Only in case of previous colonisation.
- ☐ Unknown or uncertain

f. Comments on admission screening

In case the above details do not reflect your hospital's approach correctly, please indicate further details:

5. VRE-Screening during inpatient stay

a. Which wards perform regular follow-up screening for ESBL? - *Multiple answers possible*

- ☐ Haematology/Oncology
- ☐ Intensive Care Units
- ☐ Whole hospital (only if screening approach is identical in the whole hospital)
- ☐ Unknown or uncertain

b. Which patients are screened during inpatient stay in these wards?

- ☐ All patients
- ☐ Patients with known colonisation
- ☐ Other patients with a certain risk – please indicate: ______________________________________
- ☐ Unknown or uncertain

c. How is VRE sceening performed? - *Multiple answers possible*

- ☐ Rectal swab
- ☐ Stool sample
- ☐ Rectal swab or stool sample
- ☐ Perianal swab
- ☐ Throat swab
- ☐ Urine sample
- ☐ Other, please indicate: ___________________________________
- ☐ Unknown or uncertain

d. How often is VRE follow-up screening performed?

- ☐ Once per week
- ☐ Shortly before discharge
- ☐ Other frequency, please indicate: ____
- ☐ Unknown or uncertain

e. Comments on follow-up screening

In case the above details do not reflect your hospital's approach correctly, please indicate further details:

6. Admission screening for MRSA

a. Which wards perform admission screening for MRSA? - *Multiple answers possible*

- ☐ Haematology/Oncology
- ☐ Intensive Care Units
- ☐ Whole hospital (only if screening approach is identical in the whole hospital)
- ☐ Unknown or uncertain

b. Which patients are screened on admission to these wards?

- ☐ All patients
- ☐ Patients with known colonisation
- ☐ Other patients with a certain risk – please indicate:
  ____
- ☐ Unknown or uncertain

c. How is MRSA sceening performed? - *Multiple answers possible*

- ☐ Nasal swab
- ☐ Groin swab
- ☐ Axilla swab
- ☐ Perianal swab
- ☐ Other, please indicate: ___________________________________
- ☐ Unknown or uncertain

d. How is MRSA testing performed?

- ☐ Only rapid test (PCR)
- ☐ Only culture
- ☐ Rapid test and culture
- ☐ Unknown or uncertain

e. When is MRSA admission screening performed?

- ☐ Within 48 hours of admission
- ☐ Within 72 hours of admission
- ☐ Other, please indicate: ____
- ☐ Unknown or uncertain

f. Are patients pre-emptively put under contact precautions until results of the screening are available?

- ☐ Yes
- ☐ No
- ☐ Only in case of previous colonisation.
- ☐ Unknown or uncertain

g. Comments on admission screening

In case the above details do not reflect your hospital's approach correctly, please indicate further details:

7. MRSA-Screening during inpatient stay

a. Which wards perform regular follow-up screening for ESBL? - *Multiple answers possible*

- ☐ Haematology/Oncology
- ☐ Intensive Care Units
- ☐ Whole hospital (only if screening approach is identical in the whole hospital)
- ☐ Unknown or uncertain

b. Which patients are screened during inpatient stay in these wards?

- ☐ All patients
- ☐ Patients with known colonisation
- ☐ Other patients with a certain risk – please indicate: ______________________________________
- ☐ Unknown or uncertain

c. How is MRSA sceening performed? - *Multiple answers possible*

- ☐ Nasal swab
- ☐ Groin swab
- ☐ Axilla swab
- ☐ Perianal swab
- ☐ Other, please indicate: ___________________________________
- ☐ Unknown or uncertain

d. How is MRSA testing performed?

- ☐ Only rapid test (PCR)
- ☐ Only culture
- ☐ Rapid test and culture
- ☐ Unknown or uncertain

e. How often is MRSA follow-up screening performed?

- ☐ Once per week
- ☐ Shortly before discharge
- ☐ Other frequency, please indicate: ____
- ☐ Unknown or uncertain

f. Comments on follow-up screening

In case the above details do not reflect your hospital's approach correctly, please indicate further details:

8. Contact precautions

a. Please indicate in case of detected colonisation with which pathogens contact precautions are applied:

	ESBL	VRE	MRSA	No contact precautions	Unknown
**Haematology/Oncology**	☐	☐	☐	☐	☐
**Intensive Care Units**	☐	☐	☐	☐	☐
**Whole hospital (with identical precautions)**	☐	☐	☐	☐	☐

b. Which elements are part of contact precautions for ESBL? - *Multiple answers possible*

☐ Single room
☐ Cohorting in case of unavailability of single rooms
☐ Wearing of gowns
☐ Wearing of gloves
☐ Wearing of masks
☐ Wearing of hair cover
☐ Other, please indicate: ______________________________
☐ No contact precautions
☐ Unknown or uncertain

c. Conditions under which ESBL colonised patients are allowed to leave their room? - *Mehrfachantworten möglich.*

☐ Leaving the room not allowed
☐ Only in case of urgent diagnostics
☐ The patient can leave the room when certain precautions are taken (see below)
▫ Other, please indicate: ______________________________

Precautions for patients when leaving the room:

☐ Wearing of gowns
☐ Wearing of gloves
☐ Wearing of masks
☐ Wearing of hair cover
☐ Other, please indicate: ______________________________
☐ No precautions
☐ Unknown or uncertain

d. Which elements are part of contact precautions for VRE? - *Multiple answers possible*

- ☐ Single room
- ☐ Cohorting in case of unavailability of single rooms
- ☐ Wearing of gowns
- ☐ Wearing of gloves
- ☐ Wearing of masks
- ☐ Wearing of hair cover
- ☐ Other, please indicate: ____
- ☐ No contact precautions
- ☐ Unknown or uncertain

e. Conditions under which VRE colonised patients are allowed to leave their room? - *Mehrfachantworten möglich.*

- ☐ Leaving the room not allowed
- ☐ Only in case of urgent diagnostics
- ☐ The patient can leave the room when certain precautions are taken (see below)
- ☐ Other, please indicate: ____

Precautions for patients when leaving the room:

- ☐ Wearing of gowns
- ☐ Wearing of gloves
- ☐ Wearing of masks
- ☐ Wearing of hair cover
- ☐ Other, please indicate: ____
- ☐ No precautions
- ☐ Unknown or uncertain

f. Which elements are part of contact precautions for MRSA? - *Multiple answers possible*

- ☐ Single room
- ☐ Cohorting in case of unavailability of single rooms
- ☐ Wearing of gowns
- ☐ Wearing of gloves
- ☐ Wearing of masks
- ☐ Wearing of hair cover
- ☐ Other, please indicate: ____
- ☐ No contact precautions
- ☐ Unknown or uncertain

g. Conditions under which MRSA colonised patients are allowed to leave their room? - *Multiple answers possible .*

- ☐ Leaving the room not allowed
- ☐ Only in case of urgent diagnostics
- ☐ The patient can leave the room when certain precautions are taken (see below)
- ☐ Other, please indicate: ____________________________________

Precautions for patients when leaving the room:

- ☐ Wearing of gowns
- ☐ Wearing of gloves
- ☐ Wearing of masks
- ☐ Wearing of hair cover
- ☐ Other, please indicate: ____
- ☐ No precautions
- ☐ Unknown or uncertain

h. Comments on contact precautions

In case the above details do not reflect your hospital's approach correctly, please indicate further details:

9. Further measures

a. Do you perform MRSA eradication (i.e. with Mupirocine) in case of MRSA colonisation?

- ☐ Yes
- ☐ No
- ☐ Only for certain patients, please indicate: ____
- ☐ Unknown or uncertain

b. Do patients with ESBL-detection in urine get a urinal catheter? (This approach is practiced in some hospitals in order to reduce the risk of transmission)

- ☐ Yes, in case of urinal tract infection
- ☐ Yes, in case of colonisation
- ☐ No
- ☐ Unknown or uncertain

c. Is the choice of empirical antibiotic treatment in the case of supposed infection adapted to the colonisation status? - *Multiple answers possible*

- ☐ Yes, in case of colonisation with ESBL
- ☐ Yes, in case of colonisation with VRE
- ☐ Yes, in case of colonisation with MRSA
- ☐ No
- ☐ Unknown or uncertain

End of survey

Thank you for completing the survey. If you were not able to answer all questions, we can contact one of your colleagues. Please provide the contact details below.

Name of colleague: ____

Department of colleague: ____

How can we contact this colleague? ___________________________________

(If possible, please provide the E-Mail adress)

Feedback: Do you have any comments or feedback on our survey?

Thank you!
